# Impact of Visual Elements of Tobacco Packaging on Health Risk Perceptions of Youth Groups

**DOI:** 10.3390/ijerph192114097

**Published:** 2022-10-28

**Authors:** Yanlong Guo, Yinrui Xu, Denghang Chen

**Affiliations:** 1 Social Innovation Design Research Centre, Anhui University, Hefei 203106, China; 2 Anhui Institute of Contemporary Studies, Anhui Academy of Social Sciences, Hefei 203106, China; 3 Department of Science and Technology Communication, University of Science and Technology of China, Hefei 203106, China; 4 Research Center for Science Communication, Chinese Academy of Sciences, Hefei 203106, China

**Keywords:** tobacco packaging, visual elements, youth groups, risk perception

## Abstract

Tobacco products are hazardous to public health and are one of the greater public health threats facing the world to date. Although international research on tobacco packaging has been thorough and comprehensive, the risk perception of visual elements in tobacco packaging varies by country, race, and smoking status. Therefore, the study aimed to investigate the risk perceptions of visual elements in tobacco packaging among young and middle-aged people in selected cities in China. This study used a questionnaire to construct an index system for visual elements of tobacco packaging and used it to design a related questionnaire. Our group conducted an online questionnaire survey among 296 young people (18–44 years old) in selected cities in China between 16 June and 26 June 2022. The results of the influence of visual elements of tobacco packaging on the perception of tobacco health risks in the youth group were analyzed by SPSS 26.0. A chi-square test analysis yielded differences in the perception of tobacco package color among youths with different smoking status. A linear regression analysis revealed that age group and visual elements were significant, and five groups of visual element comparisons had an effect on the youth group. First, there were differences in the perceptions of tobacco products among participants with different smoking status. Secondly, the more youthful the respondents were, the greater the probability that they were able to identify that the picture fitness warnings had a greater probability of making them conscious of the fitness dangers of smoking (*p* < 0.05). The older the participants, the greater the probability that the textual content fitness warnings made them conscious of the fitness risks of smoking (*p* < 0.05). Third, the percentage of health warnings did not make a good-sized impact for the youth groups (*p* > 0.05). Fourth, the more youthful the participant, the greater the probability that cigarette products with whole brand images would appeal to buyer(*p* < 0.01).

## 1. Introduction

Tobacco products are dangerous to public fitness and have been recognized by the World Health Organization (WHO) as one of the principal public fitness threats affecting the world [1,2]. Tobacco package design has been a key area of focus for the tobacco industry since Framework Convention on Tobacco Control (FCTC) and the International Tobacco Control Strategy (ITSC). Tobacco companies use package design to communicate brand image and have an effect on customer perceptions of tobacco flavor and strength [3,4,5]. The 2003 World Health Assembly established regulations related to tobacco packaging to ensure that packaging and labeling of tobacco products are not advertised in a false or misleading manner, and to encourage packaging standardization [6]. Packaging plays a key role in product promotion by improving the image of the manufacturer, increasing the association of high-quality products, and adding appeal [7]. Conversely, limiting the color and brand image of tobacco packaging reduces product appeal and positive associations [8,9]. Numerous scholarly studies have demonstrated that plain packaging will increase memory for health warning messages, which can enhance awareness of health risks and promote cessation behaviors [10,11,12,13]. Photo warnings are more likely to elicit better cognitive and emotional responses than plain text warnings and are more effective in increasing people’s willingness to not smoke and to quit [14,15,16]. Some scholars have also shown that removing positive representational characters from packaging can reduce the appeal of brands and reduce the chances of young women trying smoking [17]. In conclusion, the visual composition of tobacco packaging can promote or discourage use behavior among youth groups.

Risk perception of tobacco packaging refers to people’s perceptions of the health hazards of tobacco products after viewing various tobacco packaging in the marketplace [18,19]. Tobacco packaging is an important medium for developing perceptions of the harmful health risks of tobacco among youth groups and is particularly attractive to adolescents and young adults in the formative years of smoking behavior. Perceptions of health risks are also influenced through brand image and package color [3,20,21]. The lighter hues of the equal coloration and the percentage of white space on the packaging can manipulate customer perceptions of health risks [22]. Several studies have confirmed that brand names have a greater impact on adolescents’ preference to try smoking. Tobacco packaging with contrasting colors and bold graphic elements increased the desire of adolescents to try such cigarettes. Participants believed that such packaging was designed for young people [23,24]. Mercincavage et al. investigated Marlboro cigarettes with a changed brand image, in which participants’ misconceptions about nicotine and tar persisted [25]. He also showed through his study that both smokers and non-smokers recognized greater health risks and fewer misconceptions (compared to no content) after reading health warnings [26]. In the context of the international promotion of plain tobacco packaging, tobacco companies are increasingly relying on brand naming and marketing of the cigarettes themselves to differentiate their products. Attractiveness is increased by changing some visual elements of tobacco packaging [27,28,29]. All of the above studies show that the visual elements of tobacco packaging are closely related to people’s perceptions of health risks. Brand image and color are the two visual elements that have a greater degree of influence.

To effectively control the tobacco epidemic, the World Health Organization (WHO) has proposed six tobacco control strategies: MPOWER (M: Monitoring tobacco use and prevention policies; P: Protecting people from the dangers of tobacco smoke; O: Providing help to quit smoking; W: Warn about the dangers of tobacco; E: Ensure bans on tobacco advertising, promotion and sponsorship; R: Raise taxes on tobacco). The W stands for Warning of Tobacco Hazards. Health warning labels on cigarette boxes are an important part of tobacco companies’ efforts to warn smokers about the health hazards of tobacco [30].The use of text or picture health warning labels on tobacco boxes can serve as a good warning to people who are trying to smoke for the first time [31]. It has been shown that plain tobacco packaging with pictorial health warnings successfully reduces the attractiveness of tobacco, and pictorial warning messages are easier to understand. Text warnings, on the other hand, effectively present health risks and also partially reduce the misleading effect of tobacco packaging on the hazards of smoking [32,33]. Skurka et al. found that pictorial health warnings had a greater negative effect than text-only health warnings but did not improve respondents’ perceptions of health risks [34]. Health warning labels, as one of the important visual elements in tobacco packaging, are of high research value.

At present, there are few studies on the influence of tobacco packaging on health risk views of different populations in China [35]. My group created the relevant questionnaire on the online questionnaire platform from 16 June to 26 June 2022. The questionnaire was administered to young people aged 18 to 44 years. To explore the extent to which the youth group perceives the health risks of different visual elements of tobacco packaging. Although the international research on tobacco packaging has been more in-depth and comprehensive, the risk perception of some visual elements in tobacco packaging varies among people of different races and smoking status in different countries. Therefore, this learns about goals to inspect the health risk perceptions of visual elements in tobacco packaging among young people in selected cities in China.

## 2. Materials and Methods

### 2.1. Establish a Visual Element Index System for Tobacco Packaging

The visual elements were extracted with reference to the global theoretical literature on tobacco packaging research. Combined with the tobacco packaging norms proposed by the World Health Organization, the potential factors affecting the public’s perception of the health hazards of tobacco packaging were summarized, and a visual element index system for tobacco packaging was constructed. The system was also used to look at the effect of visible elements of tobacco packaging on chance perceptions of youth groups. The index system consists of three levels: goal layer, standard layer and index layer. The first layer is the target layer, i.e., tobacco packaging visual elements; the second layer is the standard layer, including brand image, package color, health warning logo style, and health warning logo ratio.

The index reference for brand image was taken from a mall intercept study conducted by Hammond et al. in 2009 among adult smokers and non-smokers in Canada. This study verified that perceptions of health risks were influenced by brand image and package color [36]. The use of package color as an indicator comes from the results of a 2009 study by Hammond et al. This study verified that tobacco package color was also associated with false beliefs about tar content and health risks: lighter-colored packages were considered less dangerous [37]. Lempert L K et al. 2017 examined how tobacco companies use package color to manipulate purchaser perceptions of cigarette flavor. Experimental studies using eye tracking have verified that tobacco companies use the color of cigarette packages to control not only smokers’ brand preferences and perceptions of harm, but also their perceptions of cigarette flavors [38]. The reference indicator for the style of health warning signs comes from a 2009 study by Fong G T et al. This study suggests that images and photographs may have an advantage over text-only messages in telling the story of health risks. Pictorial health warnings were more likely to convince people of the risks of smoking than textual health warnings and were more likely to motivate people to give up smoking [39]. However, there are also studies with conflicting results. A study by Pepper JK et al. showed that the non-smoking adolescent population was not as agreeable to the effect of picture health warnings in reducing smoking behavior [40]. Sabbane LI et al. showed that picture health warnings were effective in reducing the intention to smoke among Canadians, but not among Americans [41]. The degree of perception of health warning style also varies between populations across countries. The indicator reference for the proportion of health warning labels comes from Maansi Bansal-Travers et al. They examined the impact of the percentage of fitness warning labels on the perceived health risks of tobacco packaging among adult smokers and nonsmokers in America. The study confirmed that when asked which style of warning was most effective, the majority of participants chose the larger proportion of graphic health warning labels [42]. Germain et al. in 2010 showed that reducing brand image, while introducing larger warnings, reduced the perception of packaging and smoking experience for both non-smokers and smokers [9].

The third layer is the evaluation factor layer, which is the embodiment of the second standard layer. Includes nine evaluation factors such as complete brand image, restricted brand image, no brand image, solid color base, colored base, no color base, text health warning logo, picture health warning logo, no health warning logo, and percentage of health warning logo. Summarize the indicators defined in the relevant literature and replacing the relevant indicators with letter symbols. The names of the relevant indicators are replaced by letter symbols, as detailed in Table 1.

### 2.2. Questionnaire Title Design

The questionnaire was based on the tobacco packaging norms developed by the World Health Organization [43]. The content included demographic characteristics, comparison of brand images of cigarette packets, comparison of tobacco packaging colors, attitudes expressed on different tobacco health warning logo styles and attitudes toward the proportion of health warning logos on different cigarette packets.

The questionnaire questions were designed around four areas. The survey of perceived health risks of cigarette packets included: the textual content or picture of the packet would make you feel more strongly that smoking is unsafe to your health, the color of the packet would make you feel that its cigarette tar content is higher, the proportion of health warning labels would generally make you consider the health risks of smoking, and the brand image would make you consider buying the cigarette. Questionnaire participants had been requested to evaluate every packing box along three dimensions: (1) brand attractiveness (“compared to the same brand, cigarettes with a different brand image are more attractive to people your age”); (2) tar content (“compared to the same package color, do you think these cigarettes have a higher tar content is higher”); (3) health risks (“compared with the same brand of cigarettes, the greater the proportion of its fragrance fitness warning labels can make you aware of the fitness dangers of smoking” “compared with the same brand of cigarettes, the text or pictures of its cigarettes can make you aware of the fitness dangers”). Each of these dimensions used to be rated after viewing 12 cigarette packets, with a scale of extremely disagree (1) to extremely agree (9). The statistical results have been used to analyze the extent to which visual elements below one indicator layer in tobacco packaging have different effects on risk perceptions among youth groups, and to predict trends in the influence of visual elements in tobacco packaging design.

Question comparison design: complete brand image vs. restricted brand image (other indicators remain unchanged), complete brand image vs. no brand image, restricted brand image vs. no brand image, solid color packaging vs. color packaging, solid color packaging vs. no color packaging, color packaging vs. no color packaging, text health warning logo vs. picture health warning logo, text health warning logo vs. no health warning logo, picture Health warning logo vs. no health warning logo. Health warning logo ratio 30% vs. health warning logo ratio 50%, health warning logo ratio 50% vs. health warning logo ratio 75%, health warning logo ratio 30% vs. health warning logo ratio 75%.

### 2.3. Investigation Subjects

The survey was selected from a group of young people between the ages of 18 and 45, and the questionnaire was distributed online to participants with Chinese IP addresses, including smokers and non-smokers. As the youth group is the main workforce and producer of the country, tobacco affects the health of many young people [44]. The smoking charge among human beings aged 15 years and older in China used to be 26.6% in 2018, and extra than 1 million human beings lose their lives to tobacco each annum [45]. Chinese scholars have conducted little research on controlling and preventing the use of tobacco products in youth groups and want to improve the appearance of tobacco packaging to reducing purchasing and the use of tobacco by younger people at the source.

### 2.4. Survey Methodology

As of December 2020, it is estimated that the number of web users in China reached 989 million, of which roughly 80% are younger humans aged 10–44 [46]. Therefore, online surveys are an easy way to reach the younger population. The questionnaire survey was conducted using a Chinese questionnaire website, Questionnaire Star (www.wjx.cn (Between 16 June and 26 June 2022)), and online questionnaires were collected from May 2022 to June 2022. A total of 322 online questionnaires were collected in the end, and 26 of the questionnaires collected were invalid because of unqualified age groups and too short response time. The overall questionnaire efficiency was 91.92%.

A self-administered online questionnaire was used, and the questionnaire was quality controlled and collected through the questionnaire platform. The questionnaire used a 9-point scale format to set attitude questions. The cigarette box packaging used in the questionnaire refers to the more common brands of tobacco in China, which were imitated according to the purpose of the study.

**Figure 1:**
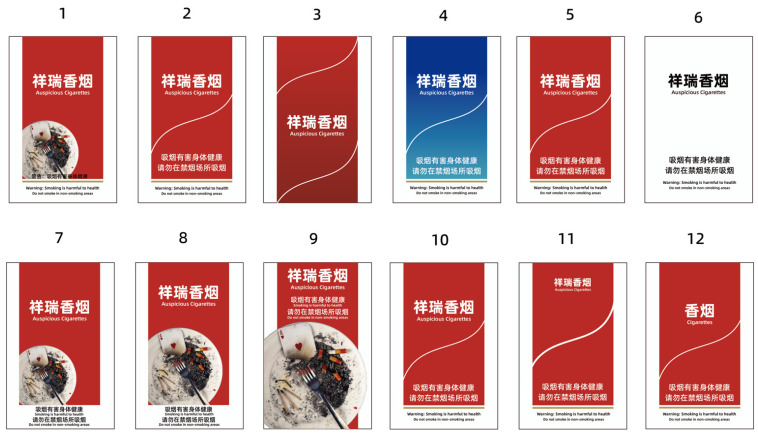
Picture of the cigarette box packaging used in the questionnaire survey.

The current imitation cigarette box packaging chooses tobacco brand cigarette box packaging as the basis. Made according to the WHO Framework Convention on Tobacco Control (FCTC) tobacco packaging requirements for cigarette boxes. For standardized cigarette box packaging refer to international cases of their own design, cigarette box brand identity and warning labels each occupy 50% of the box area. The background color of the standardized cigarette box is a solid color, and the brand names are displayed on the cigarette box packaging in standard colors and fonts only. The brand image mimics the design of a marketed tobacco brand with a fictitious brand name. As shown in Figure 1 below, Packages 1–3 is the control group. Packages 1 and 2 were designed using the FCTC packaging requirements and reference to the current packaging status in China, while Package 3 completely removes the health warnings and displays only the brand image. Packages #4–6 were the control group, and package #4 was chosen to use blue gradient packaging, as light gray and blue packaging is typically perceived by smokers as having less tar and a milder flavor than red packaging [47]. No. 5 packaging used the more popular red color of Chinese tobacco packaging, red or dark packaging is more likely to give smokers the feeling of rich, strong tobacco [48]. Package 6 mimics some of the international standardized tobacco packaging design, removing the color but retaining the brand image and health warnings. Some studies have shown that white and light-colored packages of cigarettes taste lighter and are less harmful [49]. Packages 7–9 was controls, drawn to look like 30%, 50%, and 75%, respectively. The studies have shown that packages with more branding elements remain popular, even though they also contain 50% health warnings, but with 75% warnings are more likely to trigger quit-related behaviors [50]. Packages #10–12 were the control group with full brand, restricted brand image and no brand image, respectively. Removing the brand image from the package was effective in achieving discouragement of tobacco purchase [51].

## 3. Results

### 3.1. Analysis of Basic Information of the Questionnaire

According to Table 2, the numerical traits of the demographic variables can be seen, which can reflect the distribution of the respondents of this survey. The results of the frequency analysis of the gender and age of the respondents can be viewed that the distribution essentially meets the necessities of the survey. Among them, the gender survey outcomes can be viewed in the male accounted for 36.49%, female accounted for 63.51%; in the age survey effects can be viewed mainly in the 18–25 years of age group, a complete of 204 people, accounting for 68.92%, followed by the 26–44 years of age group, a total of 92 people, accounting for 31.08%, smoking groups accounted for 18.58%, non-smoking groups accounted for 81.42%. The current questionnaire survey is mainly the evaluation of the Chinese urban youth group. The main sources of the answer addresses were Anhui with 53.5%, Shandong with 6.29% and Guangdong with 5.59%.

### 3.2. Questionnaire Reliability Test

Cronbach Alpha is a reliability take a look at performed in SPSS 26.0 to measure the interior consistency, or reliability, of an instrument or questionnaire [52,53]. It is most commonly used for questionnaires developed using multiple Likert scales, thus determining whether the scale is reliable [54]. The authors conducted a Cronbach’s reliability analysis to determine whether this questionnaire is true using SPSS, which was used to understand the reliability of the responses to the mindset scale questions; as can be considered in Table 3 below: the value of reliability was once 0.812, 0.812 > 0.7, thus indicating a very satisfactory reliability of the study facts [55]. For the “alpha coefficient of item removed”, the reliability coefficient does no longer enlarge appreciably when any problem item is deleted, as a result indicating that the problem item ought to now not be removed from the treatment.

In summary, the internal consistency reliability takes a look at of the questionnaire items yielded a Cronbach’s alpha coefficient of 0.812, which indicates good reliability and high internal consistency of the questionnaire. The study data reliability coefficient value is greater than 0.8, which together suggests high satisfactory of data reliability and can be used for in addition analysis.

### 3.3. Questionnaire Validity Test

Validity testing refers to the measurement of the validity of the questionnaire study data. Whether the results obtained through the questionnaire are true or not, and whether the respondents’ evaluation is objective or not. It is necessary to test the validity of the results of the questionnaire, and usually the validity indicators commonly used in research are Content Validity (CV) and Structural Validity (SV) [56].

The validity analysis of the questionnaire was conducted using SPSS 26.0, using exploratory factor analysis to achieve structural validity tests. According to the results of exploratory factor analysis, it is used to study the rationality of the attitude scale question design; validity analysis is used to analyze whether the question items are true and meaningful, and factor analysis, a statistical method for validity evaluation, is used to affirm the validity of the data through comprehensive analysis of KMO values, commonality, variance interpretation rate values, factor loading coefficient values, and other indicators, respectively [57].

The questionnaire validity test uses structural validity, the result of validity reflects the accuracy of the questionnaire items. The structural validity reflects the relationship between the results of the questionnaire measurement and the measured items. There are two indicators of structural validity, which are KMO value and Bartlett’s sphericity test value. The coefficient of KMO check degrees from 0 to 1, and the nearer the coefficient is to 1, the higher the validity of the questionnaire [58]. The Bartlett’s sphericity test needs to be less than 0.01. The questionnaire is imported into SPSS for analysis, the KMO value is 0.783, and the Bartlett’s sphericity test result is 0.000, which indicates that the structural validity of the questionnaire is good, and each factor is previously correlated and can be analyzed by factor analysis (Table 4). According to the significance of the sphericity check also shows that the significance of this check is infinitely shut to 0 and rejects the unique hypothesis; therefore, this questionnaire has suitable validity.

### 3.4. Cardinality Test of Smoking Status and Visual Elements

SPSS 26.0 was used to calculate the correlation between smoking status and visual elements in the study sample. The chi-square test (Table 5) showed that the response of people with different smoking status to the color of colored tobacco packaging compared to solid-colored tobacco packaging in this sample data was significant (*p* = 0.006 < 0.05). The cross tabulation (Table 6) shows that the majority of the smoking group in this study thinking that the tar content material of colored tobacco packages was greater than that of solid-colored packages. The number 1 in the table represents the smoking status as smoking, 2 represents the smoking status as non-smoking. A total of 65.5% of the smoking group agreed more with the visual element of the comparison. On the contrary, most of the non-smoking group thought that the tar content of colored tobacco packages was lower than that of solid-colored packages. A total of 45.2% of the non-smoking group disagreed with this visual element, while 18.2% of the non-smoking group had a neutral attitude toward this visual element. Tobacco internal industry research documents that smokers have a rich, strong smoking perception of red and dark tobacco packaging. While smokers’ perception of mild, mellow flavors is associated with lighter colors such as light blue or silver [59]. People with different smoking status perceive tobacco packaging colors differently. While the contrast of other visual elements is not strongly significant. So only the problematic data of colored tobacco packaging compared to solid color tobacco packaging colors were selected for analysis.

### 3.5. Linear Regression Analysis of Age Group and Visual Elements

Linear regression evaluation was used to explore the relationship between the influence of visual elements of tobacco packaging on the distribution of age groups among the youth group. Whether or not there is an impact on relationship, what is the trend of impact and the degree of impact situation [60]. As can be seen from Table 7, the 12 questions in the questionnaire regarding the comparison of visual elements were used as independent variables. While the age group is used as the dependent variable, the data from the table shows that the model has R^2^ = 0.137, which potentially indicates that the 12 questions created by the questionnaire can explain the reason for the 13.7% change in the age group. The model passed the F-test, where the test result F = 3.730, *p* = 0.000 < 0. 05, which suggests that at least one of the 12 questions in this questionnaire will have an influence on the age group. In addition, the more than one cointegration test of the model determined that the VIF values in the model are all much less than 5, indicating that there is no cointegration problem; the D-W value is 1.834, indicating that there is no pertinence between the data and the model is more ideal [61].

The regression coefficient value for cigarette packages with picture health warning labels versus text health warning labels was 0.03 (t = 1.188, *p* = 0.236 > 0.05), indicating that there was no significant relationship between the age groups of the youth groups investigated in this study. The value of the regression coefficient for cigarette packages with picture health warnings versus those without health warnings was −0.065 (t = −2.839, *p* = 0.005 < 0.01), which implies that cigarette brands with picture health warnings have a significant negative effect on the age group. The younger the youth group surveyed in this study, the more likely they are to perceive picture health warnings to be aware of the health risks of smoking. The coefficient value of the comparison question between cigarette packages with textual health warnings and those without health warnings was 0.035 (t = 2.151, *p* = 0.032 < 0.05), which implies that cigarette packages with textual health warnings have a significant positive effect on the age group. The older the participants in the formative years crew in this study, the extra likely they have been to perceive that text health warnings had been greater possibly to make them conscious of the health dangers of smoking.

The regression coefficient value for colored cigarette packs compared to solid-colored packs was 0.046 (t = 2.940, *p* = 0.004 < 0.01), implying that colored cigarette packs produced a vast positive impact relationship on age group. The older the participants in the youth group investigated in this study, the more likely they were to perceive that blue colored tobacco packages were more likely to have higher tar content than red packages. The regression coefficient of colored cigarette packs versus colorless packs used to be −0.055 (t = −3.234, *p* = 0.001 < 0.01), implying that colored cigarette packs have an extensive negative impact on age group. The younger the participants in the youth group investigated in this study, the more likely they were to perceive that blue colored tobacco packages were more likely to have higher tar content compared to colorless tobacco packages.

The question of comparison between health warning labels of different scale sizes, significant (*p* > 0.05) for all three questions, did not show significant differences. The regression coefficient value for cigarette packages with a full brand image was −0.059 (t = −2.768, *p* = 0.006 < 0.01) compared to cigarette packages without a brand. This implies that cigarette packages with complete brand image will have a significant negative correlation for the age group. The younger the participant in the youth group in this study, the more likely they would perceive that cigarette packages with complete brand image would attract him to purchase. No significant differences were found in the comparison of visual elements of other tobacco packages in the same dimension.

## 4. Discussion

First, risk perceptions of visual elements of tobacco packaging were explored among urban Chinese youth, and it was demonstrated that text and picture health warnings had been extra possibly to make youth conscious of the fitness dangers of smoking than no warnings (*p* < 0.05). However, the effect of text health warnings versus picture health warnings on the youth group was not significant, and the Chinese urban youth group did not strongly respond to picture health warnings (t = 1.188, *p* = 0.236 > 0.05), which is different from the results of international studies [14,15,16,37,38]. The older the participants, the greater the discouraging effect of text health warnings, while the younger the participants, the greater the discouraging effect of picture health warnings (Table 7). This may be related to the current status of tobacco packaging in China, as older participants were exposed to more Chinese cigarette packaging with textual warning labels and formed certain stereotypes, resulting in age differences in perception.

Second, different colored tobacco packages also produced different perceived outcomes for the youth groups. The *p*-values in the results of linear regression analysis were all less than 0.01, confirming that colored cigarette packages were more likely than colorless and solid-colored packages to make participants perceive that tobacco has higher tar content and is more harmful to health (t = 2.940, *p* = 0.004 < 0.01, t = −3.234, *p* = 0.001 < 0.01). This is similar to the conclusions reached by international scholars regarding standardized tobacco packaging [37,38]. Standardized tobacco packaging, i.e., the use of white or colorless tobacco packaging, reduces smokers’ desire to purchase. Older participants perceived colored packaging as having a higher health risk than solid-colored packaging.

Third, the brand image in tobacco packages also had a different cognitive impact on the youth group. It was confirmed that the youth group has a high recognition of the complete brand image (*p* = 0.006 < 0.01), most of them believe that they will buy cigarettes with complete brand and will not have a desire to buy unbranded cigarette packages [3,8,9,20,21,23]. The restricted brand image does not influence participants to buy compared to the complete brand image (t = −2.768, *p* = 0.006 < 0.01). Tobacco packages with the brand image removed, collectively referred to as plain packaging in international studies, extend poor perceptions and emotions about packaging and smoking [10,11,12,13]. Plain packaging additionally increases avoidance and cessation behaviors [62]. Tobacco packaging without branding reduced participants’ desire to buy and not to try tobacco.

Fourth, on the issue of comparing the proportional size of health warnings on cigarette packs, the Chinese youth group did not yield statistically significant data (*p* > 0.05). There was little difference in risk perceptions of health warning label size among the Chinese youth group. This differs from the findings of international scholars, where a larger proportional size of health warning labels caused participants to consider quitting or stopping their purchases [41,63]. First, the possible reason is that the existing health warning styles in mainland China are all text-related, and graphical health warning labels are not representative, and the size of the picture scale does not affect participants’ judgment. Second, a larger proportion of the youth group in the sample did not smoke and did not have certain risk imagery for the pictorial health warnings, and the perception of risk perception may be related to the size of the text ratio. It has also been shown that the perception of health warning style varies across countries, so it cannot be proven that larger pictorial health warning logos can lead to smoking cessation and stopping purchase behavior among the Chinese youth group.

Finally, as of early 2020, nine countries worldwide have adopted standardized tobacco packaging at both the manufacturer and retail levels, and at least 16 other countries are considering adopting this policy, making the use of standardized tobacco packaging an emerging international trend [64,65]. However, China has not yet considered adopting an internationalized tobacco packaging style, and research data could provide an empirical basis for the subsequent adoption of a standardized tobacco packaging policy in China and furnish a reference for the use of a number of visual elements in tobacco packaging.

The strength of this study is the construction of visual element indicators for tobacco packaging, which provides a practical reference for future research on tobacco packaging in China. The data sample was selected based on youth groups in urban China. There are certain limitations of the study; first, this study only investigated the reactions of some urban youth groups in China to cigarette packaging, and the scope of the study was small; second, this study investigated the subjective evaluation of cigarette packaging in the youth group, and did not use objective methods such as eye-tracking to evaluate the risk perception of tobacco packaging; further, the sample data in this study was small and influenced by geography and lifestyle habits. Finally, the imitation packages used in this study were not commercially available tobacco packages in China and may differ from the actual results.

## 5. Conclusions

The overall study showed that visual elements in tobacco packaging had a greater impact on risk perceptions among the youth group. The findings regarding health warning labels as well as brand images were generally consistent with other scholars’ findings, with picture warning labels being more effective in discouraging smoking and cigarette packages without brand images being more effective in discouraging purchase behavior among the youth group. The youth group perceived colored tobacco packages as more harmful and higher in tar content. There were no significant perceived differences in the size of health warning labels.

The Chinese urban youth group did not respond strongly to the picture health warnings, which may be related to the current state of tobacco packaging in China, as participants were exposed to Chinese cigarette warning labels that were mostly textual and formed certain stereotypes. Among the Chinese youth group, there was little difference in risk perceptions of the size of health warning labels. It may be that a larger proportion of the youth group in the sample did not smoke and did not have some risk imagination for the pictorial health warnings, and the risk perception may be related to the size of the textual proportion. The youth group has a high recognition of the complete brand image, most of them believe that they will buy cigarettes with complete brand and will not have a desire to buy unbranded cigarette packages. The reason is that in the Chinese view, the quality of cigarettes is directly related to the brand image of cigarette packs.

Today, the implementation of international standardized tobacco packaging in China is still a difficult and long-term task. The data from this study can provide an empirical basis for the subsequent adoption of a standardized tobacco packaging policy in China and inform the use of some visual elements in tobacco packaging.

## Figures and Tables

**Table 1:** Tobacco packaging visual elements index system.

Target Layer	Indicator Layer	Index Layer	Reference Sources
Visual elements of tobacco packaging that influence risk perception	**B**: Brand Image	B1: Complete Brand Image B2: Restrict brand image B3: No brand image	Hammond, D., et al. (2009) [36]
	**P**: Packaging box color	P1: Solid color base color P2: Color Base P3: Colorless base color	Hammond, D., et al. (2009) [37] Lempert, L.K., et al. (2017) [38] Fong, G.T., et al. (2009) [39]
	**H**: Health warning logo style	H1: Text health warning logo H2: Image Health Warning Logo H3: No health warning label	Barrientos-Gutierrez, I., et al. (2021) [23] Grilo, G., et al. (2021) [24]
	**W**: Health warning label ratio	W1: The proportion of 30% W2: Proportional share of 50% W3: The proportion is 75%	Germain, D., et al. (2010) [9] Mercincavage, M., et al. (2021) [26] Pepper, J.K., et al. (2013) [40]

**Table 2:** Basic information of the questionnaire.

Frequency Analysis Results				
Name	Options	Frequency	Percentage (%)	Cumulative Percentage (%)
Gender	Male	108	36.49	36.49
	Female	188	63.51	100
Age group	18–25 years old	204	68.92	68.92
	26–44 years old	92	31.08	100
Smoking status	Smoking	55	18.58	18.58
	No smoking	241	81.42	100
Highest Education	High school/junior high school and below	13	4.39	4.39
	College	26	8.78	13.18
	Undergraduate	153	51.69	64.86
	Master and above	104	35.14	100
Total		296	100	100

**Table 3:** Reliability test of the questionnaire.

Cronbach’s Reliability Analysis			
Name	Correction Term Total Correlation (CITC)	The Alpha Coefficient of the Deleted Item	Cronbach Alpha Coefficient
H2 vs. H1	0.211	0.816	0.812
H2 vs. H3	0.188	0.819	
H1 vs. H3	0.382	0.805	
P2 vs. P1	0.529	0.792	
P2 vs. P3	0.542	0.791	
P1 vs. P3	0.469	0.798	
W1 vs. W2	0.529	0.792	
W1 vs. W3	0.478	0.797	
W2 vs. W3	0.524	0.792	
B1 vs. B2	0.599	0.787	
B1 vs. B3	0.503	0.795	
B3 vs. B2	0.528	0.792	

Standardized Cronbach alpha coefficient: 0.808.

**Table 4:** Questionnaire validity test.

KMO and Bartlett’s Test		
KMO value		0.783
Bartlett sphericity test	Approximate cardinality	1375.457
	df	66
	*p* value	0

**Table 5:** Chi-square test.

	Value	Degree of Freedom	Progressive Significance (Bilateral)
Pearson Cardinal	21.275 a	8	0.006
likelihood ratio	20.246	8	0.009
Linear correlation	7.938	1	0.005
Number of active cases	296		

a. 2 cells (11.1%) have expected counts less than 5. The minimum expected count is 2.60.

**Table 6:** Cross-tabulation.

Score		1	2	3	4	5	6	7	8	9	Total
Smoking status	1	5	1	3	4	6	10	11	10	5	55
	2	30	13	27	39	44	24	22	16	26	241
Total		35	14	30	43	50	34	33	26	31	296

**Table 7:** Linear regression analysis table.

Results of Linear Regression Analysis (n = 296)									
	Non-Standardized Coefficient		Standardization Factor	t	*p*	VIF	R^2^	Adjust R^2^	F
	B	Standard Error	Beta						
Constants	2.531	0.183	-	13.804	0.000 **	-	0.137	0.1	F (12,283) = 3.730, *p* = 0.000
H2 vs. H1	0.03	0.025	0.09	1.188	0.236	1.876			
H2 vs. H3	−0.065	0.023	−0.224	−2.839	0.005 **	2.034			
H1 vs. H3	0.035	0.016	0.139	2.151	0.032 *	1.361			
P2 vs. P1	0.046	0.016	0.206	2.94	0.004 **	1.603			
P2 vs. P3	−0.055	0.017	−0.233	−3.234	0.001 **	1.704			
P1 vs. P3	−0.005	0.019	−0.02	−0.277	0.782	1.752			
W1 vs. W2	0.032	0.019	0.138	1.637	0.103	2.341			
W1 vs. W3	0.013	0.02	0.058	0.654	0.514	2.598			
W2 vs. W3	−0.028	0.019	−0.121	−1.466	0.144	2.247			
B1 vs. B2	0.024	0.021	0.09	1.152	0.25	2.014			
B1 vs. B3	−0.059	0.021	−0.216	−2.768	0.006 **	1.989			
B3 vs. B2	0.016	0.019	0.061	0.844	0.4	1.725			

Dependent variable: age group; D-W value: 1.834; * *p* < 0.05 ** *p* < 0.01

## Data Availability

The experiment data used to support the findings of this study are included in the article.
